# Analyzing Trends in Mental and Behavioral Health Support for Children: A Comprehensive Study Using National Survey of Children’s Health Database

**DOI:** 10.7759/cureus.59499

**Published:** 2024-05-02

**Authors:** Emmanuel O Ilori, Nkechi M Eziechi, Chinaza Erechukwu, Nkechi B Obijiofor, Ogochukwu Agazie, Vivien O Obitulata-Ugwu, Okelue E Okobi, Lara Aderemi, Mujeeb A Salawu, Zimakor D Ewuzie, Eberechukwu G Anamazobi, Amaka S Alozie

**Affiliations:** 1 Psychiatry and Behavioral Sciences, Garnet Health Medical Center, Middletown, USA; 2 Preventive Medicine, St. Cloud State University, St. Cloud, USA; 3 Public Health, University of East London, London, GBR; 4 Neurology, Nnamdi Azikiwe University, Awka, NGA; 5 General Physician, College of Medicine, University of Lagos, Idi-Araba, NGA; 6 Medicine, University of Nigeria, Enugu, NGA; 7 Family Medicine, Larkin Community Hospital Palm Springs Campus, Miami, USA; 8 Family Medicine, Medficient Health Systems, Laurel, USA; 9 Family Medicine, Lakeside Medical Center, Belle Glade, USA; 10 Family Medicine, University of Calgary, Calgary, CAN; 11 Medicine and Surgery, University of Ilorin College of Health Sciences, Ilorin, NGA; 12 Internal Medicine and Psychiatry, Houston Health Department, Houston, USA; 13 Psychiatry, Cygnet Hospital Harrogate, Harrogate, GBR; 14 Internal Medicine, American International School of Medicine, McDonough, USA; 15 Internal Medicine, Abia State University, Umuahia, NGA

**Keywords:** disparities, national survey of children's health database, counseling, treatment trends, behavioral health, mental health, children

## Abstract

Objective

This study aimed to explore mental and behavioral health support trends for children aged 3-17, analyzing treatment and counseling using United States data from the 2016-2020 National Survey of Children's Health (NSCH) database.

Methods

Employing a retrospective observational design, we systematically retrieved and analyzed NSCH Database data from 2016 to 2020. The focus was on understanding mental and behavioral health treatment percentages over time, specifically targeting demographic variations such as age groups, gender, race/ethnicity, and the federal poverty level percentage. Graphical representation utilized Excel, summarizing results based on aggregated data for distinct time intervals, highlighting the importance of mental and behavioral health support for children aged 3-17.

Results

The study identified significant temporal trends in mental and behavioral health treatment, revealing notable fluctuations across demographic and socio-economic variables. Of the 22,812 participants, 51.7% (CI: 50.2-53.1%, n=12,686) received treatment, exposing disparities. Gender differences were evident, with higher treatment rates in females (53.7%, CI: 51.6-55.9%, n=6,166) than males (50.1%, CI: 48.2-52.0%, n=6,520). Age-specific patterns indicated lower intervention rates in younger children (33.5%, CI: 28.6-38.8%, n=447, ages 3-5) compared to adolescents (58.1%, CI: 56.2-59.9%, n=8, 222 ages 12-17).

Conclusion

The conclusion highlights significant temporal fluctuations and pronounced demographic disparities. Findings underscore varying prevalence rates among age groups, genders, racial/ethnic backgrounds, and socio-economic status categories. This study provides valuable insights for policymakers, healthcare professionals, and researchers, informing targeted interventions to enhance mental and behavioral health support for United States children.

## Introduction

Mental and behavioral health issues in children aged 3-17 encompass a spectrum of emotional, psychological, and social challenges that impact their overall well-being [1]. These concerns include anxiety, depression, attention-deficit/hyperactivity disorder (ADHD), conduct disorders, and developmental disorders. Manifesting in diverse forms, such as behavioral difficulties, mood disturbances, or impaired social interactions, these issues significantly hinder a child's normal functioning and development [2]. Early identification and intervention are crucial for providing appropriate support, counseling, and interventions, ensuring optimal mental health outcomes as children navigate the critical stages of their formative years [3].

Behavioral Health Support is crucial for addressing mental health challenges in children, offering therapeutic interventions, counseling, and personalized strategies to enhance resilience and emotional stability [4]. Annually, approximately 20% of children experience mental disorders, with an expenditure of $247 billion for treatment. The substantial impact designates these issues as a significant public health concern, highlighting the need for effective, comprehensive strategies to address children's mental well-being [5]. The study shows that 21.8% of United States children aged 3 to 17 face common mental, emotional, and behavioral health conditions, escalating with social and relational risks [6].

The 2021 National Health Interview Survey explores mental health treatment in children aged 5-17, covering medication use, counseling, or both within the past 12 months [7]. Children's mental and behavioral health pathophysiology involves a complex interplay of genetic, environmental, and neurobiological factors [8]. Neurotransmitter imbalances, altered brain structures, and genetic predispositions contribute to conditions like ADHD and anxiety [9]. Early-life stressors impact neural development, leading to persistent mental health challenges. Understanding this pathophysiology is crucial for developing targeted interventions and promoting healthy neurodevelopment, emphasizing the importance of individualized approaches to mental and behavioral health care [10-11].

The National Survey of Children's Health (NSCH), conducted by the Census Bureau in the United States, assesses children's physical and emotional well-being, providing valuable data on healthcare access, family dynamics, and socioeconomic factors [12]. This study examines treatment and counseling trends, analyzing demographic variables (age, gender, race, poverty level) to address evolving needs in mental health. It reveals temporal fluctuations influenced by demographic and socio-economic factors, which are crucial for ensuring equitable access to children's mental health care. By scrutinizing these trends, the study aims to contribute knowledge for understanding challenges, successes, and areas for improvement in mental and behavioral health support for children.

## Materials and methods

Study design and participants

This research employed a retrospective observational design, analyzing treatment and counseling trends for mental and behavioral health support in children aged 3-17. The study utilized data from the 2016-2020 NSCH Database, a comprehensive and nationally representative dataset focusing on the health and well-being of children in the United States. The data for this study were collected from the publicly available Data Resource Center for Child & Adolescent Health. The NSCH conducted the original survey, utilizing a combination of online and mail administration methods for sampling. Randomly selected addresses across the United States were mailed instructions to access the survey online, while some households also received a paper version of the screening questionnaire.

The selected timeframe provided a recent and relevant snapshot of mental and behavioral health support trends among children aged 3-17. Datasets were systematically retrieved from the NSCH database following standardized protocols, with meticulous attention to data quality and consistency to ensure analytical accuracy. The total number of participants in the analysis was derived from the database records, including a sample count of 22,812.

Study variables

Key variables encompassed utilizing mental and behavioral health treatment and counseling services. This included types of mental and behavioral health interventions, frequency of counseling, and demographic information.

Demographic Characteristics

Variables such as age, gender, race/ethnicity, and family structure were included to examine potential disparities in mental and behavioral health support across different demographic groups.

Socioeconomic Status

Indicators such as household income were considered to elucidate the influence of socioeconomic factors on access to mental health services. The study used Federal Poverty Level (FPL) set income criteria for assistance eligibility, with 0-99% FPL indicating extremely low income, 100-199% FPL qualifying as low-income, 200-399% FPL as moderate-income, and 400% FPL or more signifying higher income, making some ineligible for need-based programs but still economically relevant.

Child Health Indicators

Information on the child's overall health status, data based on adverse childhood experiences (ACE), and data based on received treatment or not provided context for understanding the intersection between physical and mental well-being.

Data analysis

Descriptive statistics were employed to characterize the study population and provide an overview of mental and behavioral health support utilization. Frequencies and percentages were computed using Excel to describe the prevalence of counseling trends. Subgroup analyses based on demographic, socioeconomic, and child health indicators were performed to identify variations.

Ethical considerations

The Institutional Review Board (IRB) is known to view the examination of anonymized, publicly accessible data that does not contain any personal identification information as not falling under the category of human subjects research as per the standards outlined in 45 CFR 46.102. Consequently, such an analysis does not necessitate a review by the IRB.

## Results

The study included 22,812 patients during the specified time frame. Overall, the data revealed that 51.7% (CI: 50.2 - 53.1%, n=12,686) of children in the sampled population received some form of mental or behavioral health treatment or counseling.

Based on gender

In our study, which examined gender-based disparities in the treatment of mental and behavioral conditions among children, a concerning percentage did not receive adequate care. Alarmingly, the results indicated that a higher proportion of females, 53.7% (CI: 51.6-55.9%, n=6,166), received treatment or counseling compared to males, 50.1% (CI: 48.2-52.0%, n=6,520). The results also indicated that a higher proportion of males, 49.9% (CI: 48-51.8%, n=5,841), did not receive treatment or counseling compared to females, 46.3% (CI: 44.1-48.4%, n=4,285) (Table 1). This disparity underscores potential gender-specific barriers to accessing mental health support for children.

**Table 1:** Treatment and counseling rates for Children (Ages 3-17) with mental/behavioral conditions by demographic variables Note- Data presented as N (%): Sample size (percentage), C.I- Confidence Interval

Demographic characterstics	Variables		Received treatment or counseling	Did not receive treatment or counseling
Data Based on Gender	Male	N (%)	6520 (50.1)	5841 (49.9)
		C.I.	48.2 - 52.0	48.0 - 51.8
	Female	N (%)	6166 (53.7)	4285 (46.3)
		C.I.	51.6 - 55.9	44.1 - 48.4
Data based on age group	3-5 years old	N (%)	447 (33.5)	933 (66.5)
		C.I.	28.6 - 38.8	61.2 - 71.4
	6-11 years old	N (%)	4017 (46.7)	3725 (53.3)
		C.I.	44.4 - 49.1	50.9 - 55.6
	12-17 years old	N (%)	8222 (58.1)	5468 (41.9)
		C.I.	56.2 - 59.9	40.1 - 43.8
Data based on race	Hispanic	N (%)	1352 (48.7)	1152 (51.3)
		C.I.	44.4 - 53.1	46.9 - 55.6
	White, non-Hispanic	N (%)	9303 (53.1)	7318 (46.9)
		C.I.	51.6 - 54.5	45.5 - 48.4
	Black, non-Hispanic	N (%)	743 (51.9)	642 (48.1)
		C.I.	47.3 - 56.3	43.7 - 52.7
	Asian, non-Hispanic	N (%)	247 (51.8)	220 (48.2)
		C.I.	42.7 - 60.8	39.2 - 57.3
	American Indian/Alaska Native, Non-Hispanic	N (%)	104 (55.9)	76 (44.1)
		C.I.	43.6 - 67.4	32.6 - 56.4
	Native Hawaiian/Other Pacific Islander, Non-Hispanic	N (%)	21 (39.6)	17 (60.4)
		C.I.	16.0 - 69.2	30.8 - 84.0
	Multiple race, non-Hispanic	N (%)	874 (49.2)	659 (50.8)
		C.I.	43.9 - 54.5	45.5 - 56.1

Based on age groups

**Figure 1:**
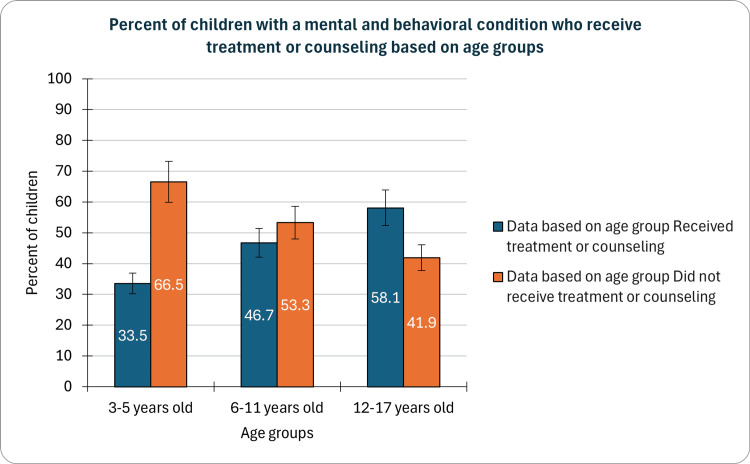
Children with a mental and behavioral condition who receive treatment or counseling based on age

In our study, which examined the prevalence of mental and behavioral conditions among children, we found that the percentage receiving treatment or counseling varied significantly across age groups. Among the youngest cohort aged 3-5 years, 33.5% (CI: 28.6-38.8%, n=447) received the intervention, while this figure increased to 46.7% (CI: 44.4-49.1%, n=4017) in the 6-11 age group. Adolescents aged 12-17 years demonstrated a further higher treatment rate at 58.1% (CI: 56.2-59.9%, n=8222) (Figure 1). These findings underscore the importance of age-specific approaches in addressing children's mental health needs, emphasizing the need for targeted interventions tailored to the developmental stages of this vulnerable population.

Based on race/ ethnicity

**Figure 2:**
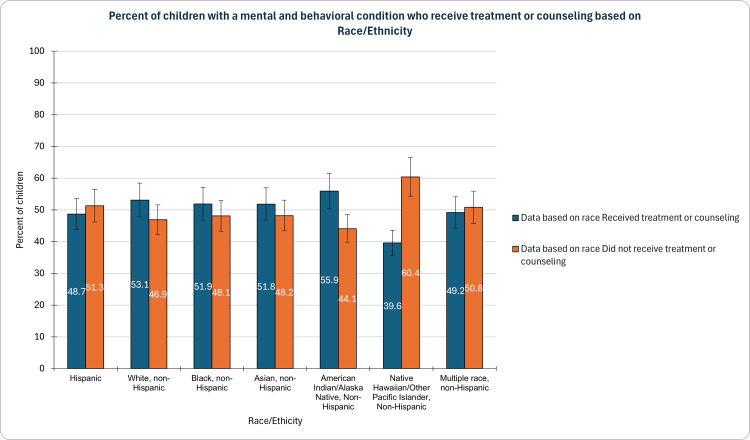
Children with a mental and behavioral condition who receive treatment or counseling based on race

Our investigation revealed significant discrepancies in the percentages of children receiving treatment or counseling, highlighting distinct patterns among various racial and ethnic groups. Notably, Native Hawaiian/Other Pacific Islander, non-Hispanic children had the lowest percentage at 39.6% (CI: 16.0-69.2%, n=21), emphasizing the pressing need for targeted interventions. Conversely, American Indian/Alaska Native and non-Hispanic children exhibited the highest rates at 55.9% (CI: 43.6-67.4%, n=104) in accessing mental health services. Hispanic and multiple races Non-Hispanic fell below 50%, with percentages of 48.7% (CI: 44.4-53.1, n=1352) and 49.2% (CI: 43.9-54.5%, n=874), respectively. Asian Non-Hispanic, Black Non-Hispanic, and White (Non-Hispanic) populations demonstrated rates of 51.8% (CI: 42.7-60.8%, n=247), 51.9% (47.3-56.3%, n=743), and 53.1% (CI: 51.6-54.5%, n=9303) respectively (Figure 2). This study underscores the urgent requirement for culturally sensitive and inclusive strategies to address mental health treatment disparities among children from diverse backgrounds.

Based on ACE

**Figure 3:**
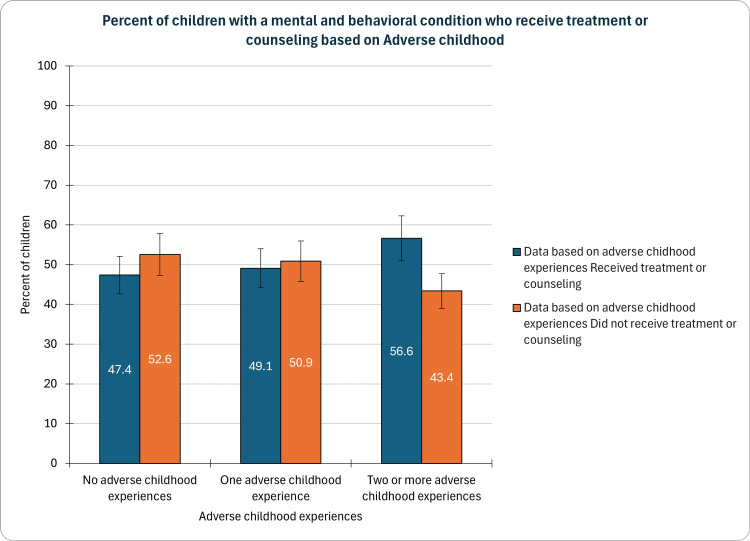
Children with a mental and behavioral condition who receive treatment or counseling based on adverse childhood experiences

In our study, which examined the prevalence of mental and behavioral conditions among children in relation to ACEs, compelling findings emerged regarding treatment and counseling rates. Notably, 47.4% (CI: 45.0-49.8%, n=4,143) of children without ACEs received such support, while those with one ACE showed a slight increase at 49.1% (CI: 46.4-51.8%, n=2,877). However, a more pronounced rise was observed among children with two or more ACEs, where the treatment or counseling rate reached 56.6% (CI: 54.2-58.9%, n=5,152); see Figure 3 below. These results underscore the escalating importance of early intervention strategies for children exposed to multiple adverse experiences, emphasizing the imperative role of targeted mental health support in mitigating the impact of childhood adversities.

Based on FPL

**Figure 4:**
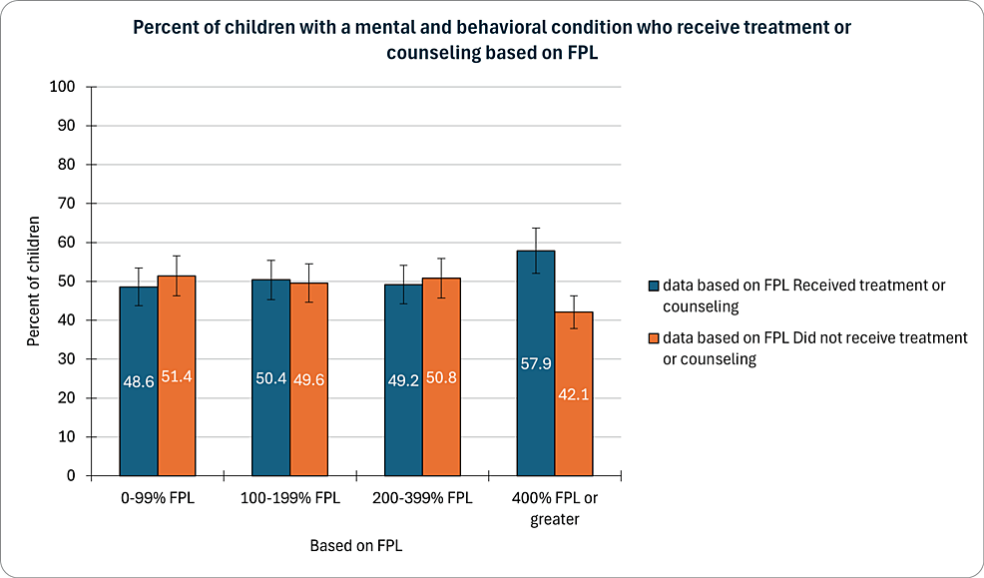
Children with a mental and behavioral condition who receive treatment or counseling based on FPL FPL- Federal poverty level, 0-99% FPL indicating extremely low income, 100-199% FPL qualifying as low-income, 200-399% FPL as moderate-income, and 400% FPL or more signifying higher income

In our study, we observed varying rates of treatment or counseling receipt among children with mental and behavioral conditions stratified by family income. Notably, 48.6% (CI: 45.0-52.2%, n=1,702) of children from families with incomes below 100% of the FPL received such services. The percentage increased marginally to 50.4% (CI: 47.3-53.5%, n=2,126) for those in the 100-199% FPL range and slightly decreased to 49.2% (CI: 46.6-51.8%, n=3,690) for families in the 200-399% FPL category. Surprisingly, the highest rate was found among children in families with incomes at or above 400% FPL, with 57.9% (CI: 55.8-59.9%, n=5,078) receiving treatment or counseling (Figure 4). These findings underscore the complex interplay between socioeconomic factors and access to mental health services for children.

Based on the received treatment

In our study, 52.4% (CI: 50.9-53.8%, n=12,314) of children with identified mental and behavioral conditions reported receiving treatment or counseling. This finding suggests a noteworthy proportion benefited from therapeutic interventions. However, a concerning 39.8% (CI: 32.2-47.9%, n=347) of children did not receive any form of treatment, indicating a substantial treatment gap (Table 2). These results underscore disparities in access to mental health care for children with such conditions. Further exploration of contributing factors, such as socio-economic status and geographical location, is warranted to inform targeted interventions. Addressing this treatment gap is crucial to ensuring comprehensive and equitable mental health support for all affected children.

**Table 2:** Treatment and counseling rates for children (Ages 3-17) with mental/behavioral conditions by socioeconomic and child health indicator variables Note- Data presented as N(%): Sample size (Percentage); CI: Confidence interval; FPL: Federal Poverty Level

Characterstics	Variables		Received treatment or counseling	Did not receive treatment or counseling
Data based on adverse chidhood experiences	No adverse childhood experiences	N (%)	4143 (47.4)	3985 (52.6)
		C.I.	45.0 - 49.8	50.2 - 55.0
	One adverse childhood experience	N (%)	2877 (49.1)	2449 (50.9)
		C.I.	46.4 - 51.8	48.2 - 53.6
	Two or more adverse childhood experiences	N (%)	5512 (56.6)	3541 (43.4)
		C.I.	54.2 - 58.9	41.1 - 45.8
Data based on FPL	0-99% FPL	N (%)	1702 (48.6)	1631 (51.4)
		C.I.	45.0 - 52.2	47.8 - 55.0
	100-199% FPL	N (%)	2216 (50.4)	1988 (49.6)
		C.I.	47.3 - 53.5	46.5 - 52.7
	200-399% FPL	N (%)	3690 (49.2)	3164 (50.8)
		C.I.	46.6 - 51.8	48.2 - 53.4
	400% FPL or greater	N (%)	5078 (57.9)	3343 (42.1)
		C.I.	55.8 - 59.9	40.1 - 44.2
Data based on received treatment or not	Yes	N (%)	12314 (52.4)	9639 (47.6)
		C.I.	50.9 - 53.8	46.2 - 49.1
	No	N (%)	347 (39.8)	450 (60.2)
		C.I.	32.2 - 47.9	52.1 - 67.8

## Discussion

This comprehensive analysis of treatment and counseling trends from the NSCH provides critical insights into the mental and behavioral health support landscape for children in the United States. The findings illuminate disparities across various demographic factors such as age groups, gender, race/ethnicity, socio-economic factors (FPL), and adverse childhood experiences, shedding light on the multifaceted challenges faced by children and their families.

Our study revealed gender-based disparities in the treatment of mental and behavioral conditions among children. Alarmingly, a higher proportion of females received treatment or counseling compared to males, indicating potential barriers that disproportionately affect boys. This finding raises questions about the factors contributing to this gender-specific gap in accessing mental health support [7,13-14]. Future research should investigate the underlying causes, considering societal expectations, stigma, or differential symptom recognition. Culturally sensitive interventions tailored to the unique needs of both genders are imperative to ensure equitable access to mental health care. 

The variation in treatment or counseling rates across age groups emphasizes the importance of age-specific approaches in addressing children's mental health needs. While adolescents demonstrated a higher treatment rate, younger children (ages 3-5) exhibited a lower percentage of receiving intervention. This underscores the need for targeted interventions tailored to the developmental stages of children, acknowledging the evolving nature of mental health challenges as they grow. Initiatives focusing on early intervention for the youngest age group are crucial to prevent long-term consequences and promote overall well-being [2,7,14].

Significant disparities were observed in the percentages of children receiving treatment or counseling across different racial and ethnic groups. Native Hawaiian/Other Pacific Islander, non-Hispanic children had the lowest rates, emphasizing the need for culturally sensitive interventions in these communities. Conversely, American Indian/Alaska Native and non-Hispanic children exhibited higher rates, suggesting potential protective factors or increased awareness. The varying percentages among different racial and ethnic groups underscore the need for targeted strategies that consider cultural nuances to ensure equitable access to mental health services [14,15].

Our study revealed compelling patterns concerning treatment and counseling rates in relation to ACEs. Notably, children with two or more ACEs demonstrated a significantly higher treatment rate, highlighting the critical role of targeted mental health support for those exposed to multiple adversities. Early intervention strategies are paramount in mitigating the impact of childhood adversities, emphasizing the need for comprehensive and trauma-informed approaches in mental health care [14].

The complex interplay between family income and access to mental health services is evident in our findings. Surprisingly, the highest rate of treatment or counseling was observed among children in families with incomes at or above 400% of the FPL. This result prompts further investigation into potential factors, such as healthcare disparities or variations in help-seeking behaviors. Addressing the disparities in mental health support across different income brackets requires a nuanced understanding of the socio-economic factors influencing access to care.

While our study indicates that a significant proportion of children with identified mental and behavioral conditions benefited from treatment or counseling, a concerning treatment gap exists, with 39.8% of children not receiving any form of intervention. Exploring contributing factors, such as socio-economic status and geographical location, is essential to inform targeted interventions addressing this gap. Comprehensive and equitable mental health support for all affected children necessitates addressing the root causes of the treatment disparities identified in our study.

While the analysis of the NCHS Database provides valuable insights, it is essential to acknowledge certain limitations that may impact the generalizability and depth of the findings. Firstly, the data rely on self-reported information from parents or caregivers, introducing response bias and relying on the accuracy of their perceptions and memories. Moreover, the survey's cross-sectional nature limits the ability to establish causal relationships or assess the longitudinal impact of treatments over time. The database predominantly captures information broadly, potentially overlooking specific nuances in treatment modalities and counseling approaches. Additionally, the study does not account for cultural and socio-economic factors that could influence access to and utilization of mental health services, limiting the generalizability of the findings across diverse populations. The limited time frame of 2016-2020 might not fully capture the dynamic changes in mental health support systems, which could have significant implications for children's mental health.

Furthermore, the survey may not encompass emerging trends or innovative interventions that have evolved in the field of child mental health after 2020. While the database offers a comprehensive overview, the absence of certain variables, such as the quality of therapeutic relationships or the presence of comorbidities, hinders a holistic understanding of the complexities involved in children's mental health treatment. Therefore, researchers and practitioners should interpret the findings with caution and consider these limitations when applying the results to inform policy, practice, and future research in the realm of children's mental and behavioral health.

## Conclusions

This analysis of mental and behavioral health support for children reveals crucial insights into treatment and counseling trends. Dynamic temporal fluctuations underscore the influence of demographic and socio-economic factors on children's mental health experiences. Disparities are evident across genders, with higher treatment rates in females, warranting exploration of potential barriers affecting boys. Age-specific trends emphasize the need for tailored early interventions, especially for younger children. Pronounced disparities across racial/ethnic backgrounds and socio-economic categories highlight the necessity for targeted strategies. Unexpectedly higher treatment rates in families with incomes above 400% of the federal poverty level challenge assumptions about socio-economic influences on mental health support. These findings advocate for nuanced, targeted interventions to address gaps in mental and behavioral health support, contributing valuable insights for policymakers, healthcare professionals, and researchers. The study promotes a more equitable approach to children's mental well-being in the United States.

## References

[1] Granlund M, Imms C, King G (2021). Definitions and operationalization of mental health problems, wellbeing and participation constructs in children with NDD: distinctions and clarifications. Int J Environ Res Public Health.

[2] Bitsko RH, Claussen AH, Lichstein J (2022). Mental health surveillance among children - United States, 2013-2019. MMWR Suppl.

[3] Colizzi M, Lasalvia A, Ruggeri M (2020). Prevention and early intervention in youth mental health: Is it time for a multidisciplinary and trans-diagnostic model for care?. Int J Ment Health Syst.

[4] Hossain MM, Purohit N (2019). Improving child and adolescent mental health in India: Status, services, policies, and way forward. Indian J Psychiatry.

[5] Johns Hopkins Bloomberg School of Public Health (2023). Study reveals a fourfold range in rates of mental health problems among U.S. children based on relational and social risks. https://publichealth.jhu.edu/2022/study-reveals-fourfold-range-in-rates-of-mental-health-problems-among-us-children-based-on-relational-and-social-risks.

[6] (2023). Mental health treatment among children aged 5-17 years: United States, 2021. https://www.cdc.gov/nchs/products/databriefs/db472.htm.

[7] Brennenstuhl H, Jung-Klawitter S, Assmann B, Opladen T (2019). Inherited disorders of neurotransmitters: Classification and practical approaches for diagnosis and treatment. Neuropediatrics.

[8] Teleanu RI, Niculescu AG, Roza E, Vladâcenco O, Grumezescu AM, Teleanu DM (2022). Neurotransmitters-key factors in neurological and neurodegenerative disorders of the central nervous system. Int J Mol Sci.

[9] Smith KE, Pollak SD (2020). Early life stress and development: Potential mechanisms for adverse outcomes. J Neurodev Disord.

[10] Brown AF, Ma GX, Miranda J (2019). Structural interventions to reduce and eliminate health disparities. Am J Public Health.

[11] (2023). National survey of children’s health. https://mchb.hrsa.gov/data-research/national-survey-childrens-health.

[12] Olfson M, Marcus SC (2010). National trends in outpatient psychotherapy. Am J Psychiatry.

[13] Ghandour RM, Sherman LJ, Vladutiu CJ, Ali MM, Lynch SE, Bitsko RH, Blumberg SJ (2019). Prevalence and treatment of depression, anxiety, and conduct problems in US children. J Pediatr.

[14] Acevedo-Garcia D, Noelke C, McArdle N (2020). Racial and ethnic inequities In children's neighborhoods: Evidence from the new child opportunity index 2.0. Health Aff (Millwood).

[15] Suh B, Luthar SS (2020). Parental aggravation may tell more about a child's mental/behavioral health than adverse childhood experiences: Using the 2016 National Survey of Children's Health. Child Abuse Negl.

